# Oral Hygiene and Lifestyle in Disadvantaged Schools in North-Eastern Hungary

**DOI:** 10.1016/j.identj.2025.02.013

**Published:** 2025-03-22

**Authors:** Ildikó Faragó, Tímea Egri, Mihály Kovács, Andrea Rucska

**Affiliations:** Faculty of Humanities, University of Miskolc, Miskolc, Hungary

**Keywords:** Disadvantage, Lifestyle, Health behavior, Oral hygiene

## Abstract

**Introduction:**

Oral hygiene and the development of good health habits play an important role in maintaining overall health and achieving a good quality of life, thereby influencing self-confidence and psychological and somatic well-being. The aim of our research was to draw attention to the poor oral hygiene indicators of marginalised and disadvantaged groups and to look for correlations between different health behaviours and lifestyles.

**Methods and data:**

Our research covered the prevalence of dental caries (decayed, missing, and filled teeth [DMFT] value), their dental visits and brushing habits, their lifestyle, psychosomatic state, and self-evaluation.

**Results:**

Sample characteristics: n = 429 (318 primary school students + 111 high school students), mean age 11.5 (SD:3.2) years. One-third of the students lived in overcrowded, uncomfortable homes, 33.3% of which had no piped water supply. Nearly 40% of students reported that there was a dentist in their village, but 73% only visited a dentist when they had a toothache. Nearly half of students brush their teeth every morning and evening, but only a third receive more than 1 toothbrush a year. Many do not even have a toothbrush or only get one if their family can afford it. More than two-thirds of students consume sugary drinks and snacks after brushing their teeth in the evening. A high percentage (51.6%) were found to have acute gingivitis, and 47.8% of students were diagnosed with chronic gingivitis. The DMFT value was high at 6 (SD: 3.2), with a significant positive correlation between these values and post-toothbrush evening meals (*p* < .019). A total of 24% of the study group consumed alcohol regularly, and there was a high prevalence of smoking. The DMFT index showed a significant correlation (*P* < .036) with alcohol consumption.

**Conclusion:**

The cumulatively disadvantaged youngsters’ oral hygiene and health behavioural habits show serious arrears, where unmotivated family background, low income, lack of dental education, and unavailable health care/preventive services all play an important role.

## Introduction

According to data from the Hungarian Central Statistical Office (KSH), the number of people living in extreme poverty in Hungary is significant, with their geographical distribution showing great inequalities. In certain regions, poverty is particularly severe, and opportunities for those living there to escape these conditions have further diminished in recent years due to events like the pandemic.^1^ This situation is exacerbated by limited access to health care services, lack of motivation, low life expectancy, and numerous other social and societal factors that critically influence the life prospects of residents in these areas.^2^

Our research was conducted in Northern Hungary, 1 of the 3 most disadvantaged regions of the country, where we examined the oral health of the population and its social impacts. In Hungary, poverty levels are determined based on subsistence calculations by the Hungarian Central Statistical Office (KSH). Poverty, unemployment, old age, disability, housing difficulties, and social exclusion are all risk factors that impose long-term burdens on every layer of society.

In the European Union, poverty is measured using a standardized indicator called the “At Risk of Poverty or Social Exclusion” (AROPE) rate. This metric determines the proportion of people at risk of social exclusion based on three indicators. The most commonly used of these is “relative income poverty”, which refers to the proportion of individuals living in households where the net income does not exceed 60% of the median income.^3^^,^^4^ The other two indicators provide insights into the degree of material deprivation and the intensity of labor market exclusion faced by those affected.^5^

**Fig. 1:**
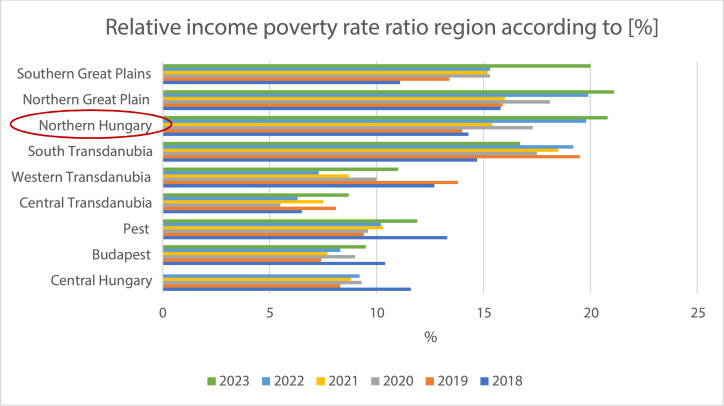
Changes in the relative income poverty rate by region and settlement type [%], 2018-2021.^4^

In Hungary, 17.7% of the total population is affected by poverty and social exclusion. However, in Northern Hungary, these problems impact every fourth household, with residents in this region experiencing deprivation at twice the national average rate^6^ (Figure 1).

**Fig. 2:**
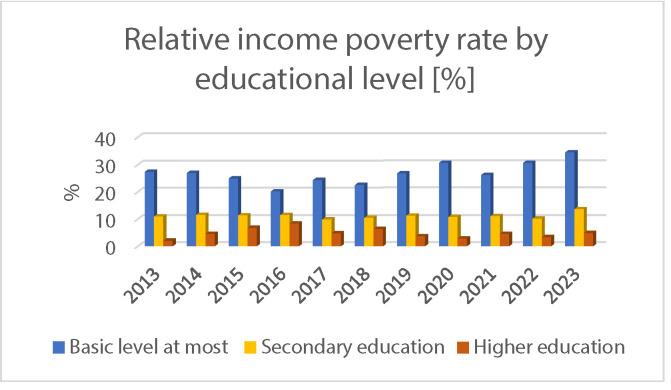
Relative income poverty rate by educational level in Hungary 2018-2021 (%).^4^

Extreme poverty is further characterized by a combination of factors, including low levels of education, segregation, unemployment, a high proportion of large families, and a significant likelihood of reproducing disadvantages across generations. In their analysis published in the book *Equal Opportunities in Today's Hungary* (2013), Tibor Cserti Csapó and Anna Orsós highlighted that among those living in extreme poverty, the “youthful face of poverty” is particularly striking; according to contemporary data, 30% of individuals living in poverty belonged to the 0 to 17 age group^7^ (Figure 2).

The north-eastern Hungarian region is considered a disadvantaged area in Hungary from several points of view.^8^ The concept of disadvantage includes individuals, groups, and communities who—to varying degrees—differ from the average and from the “well-being” of the given society. They are disadvantaged not only because of low income and lower levels of educational attainment but also because of the lack of suitable housing conditions. As a consequence, not only their biological-somatic but also their intellectual and spiritual development suffers, making them even more vulnerable. They are also heavily influenced by a poor cultural background, health reasons, lack of use of health services, inadequate family structure, and a dysfunctional family situation.^9^

From previous years' reports, we know that 17.7% of the entire population in Hungary lives in poverty and social exclusion. In Northern Hungary, every fourth household is affected by these, and here, twice as many people live in poverty than the national average.^6^ All of this is also connected to the fact that almost 100% of the students we examined are from the Roma population.

The situation of the Roma population is particularly difficult, as in their case, they also have to deal with the problems of being a minority and living in deep poverty. According to the Roma strategy of the European Union, “Gypsyism and poverty appear in the same group of issues, therefore the problems of Gypsyism listed in the preamble are actually the problems of poverty”.^10^

In disadvantaged populations, Roma children are overrepresented, and there is a clear overlap between the geographical distribution of the Roma population and the locations where the number of disadvantaged students is high. Two-thirds of disadvantaged children are classified as at-risk by child welfare services, with the largest numbers found in Borsod-Abaúj-Zemplén (B-A-Z) County and Hajdú-Bihar County.^11^

According to 2016 data from the Hungarian Central Statistical Office (KSH), unemployment significantly exceeded the national average in the villages included in the study. Additionally, the proportion of children in the total population was twice the national average at 24.8%.^8^

In settlements with such population compositions, processes markedly differ from national demographic and social trends. These areas exhibit characteristics similar to those of third-world countries, such as a youthful age structure, high illiteracy rates, low healthy life expectancy, and elevated levels of social anomie. For this reason, creating opportunities for health equity through preventive and educational activities is of critical importance on a societal level.^12^

In this study, through their oral health, we summarize the lifestyle, oral hygiene, and dental visitation habits of primary and secondary school students living in this region in the light of their socioeconomic background. In the course of the research, we examined the health status and health behavior habits of these children (of which almost 100% are Roma children), as well as the possibilities for them to use health services, including preventive care. We also looked into whether going to a higher-level school and living in a big city changed their health behavior habits and whether their oral health improved as a result.

We chose to explore these areas because the health indicators in Hungary are considered to be bad, and even within that, the health indicators of those living in deprived and disadvantaged populations can be a lot worse. Compared to the average age in the European Union, for example, Hungarian men with the lowest education live an average of 12 years less than the most educated, and for women, the difference is more than 6 years compared to the EU average.^13^ We are not doing well in terms of various risk factors either; it is estimated that half of all deaths in Hungary can be attributed to behavioral risk factors, including poor diet, smoking, alcohol consumption, and low physical activity. This rate is much higher than the EU average of 39%. Regarding the risk of poor dietary habits, the rate in Hungary is 28%, compared to 18% in the EU.^13^

Harmful health behavior habits are of particular importance in cumulatively disadvantaged groups, since we know that among them there is a higher percentage of diseases that are common in Hungary anyway, such as cardiovascular diseases, respiratory diseases (asthma, lung tumors), oral cavity tumors, and dental emerging problems. For example, 66.7% of Roma people who are over the age of 19 years already suffer from some kind of chronic illness.^14^

As we know, the family microenvironment heavily influences sociocultural behavior and an individual's relationship to health; however, in these populations, it is difficult to approach the family microenvironment with traditional methods. That is why we can only achieve results through the effective cooperation of specially trained teachers, social workers, and preventive health professionals.

Based on all of the above, we can say that it is not an easy task to examine the situation and come up with a solution for the population we are looking at—not only because of deep poverty, hopelessness, and the presence of various health-damaging risk factors, but also because this population cannot be influenced by traditional means of communication and educational tools. If we add to this the fact that in many cases there are also some specific educational needs, or they also struggle with behavior and integration disorders, further tests on their personality and self-evaluation can also be helpful and justified.

These prospects are not attractive for young, newly graduated health professionals: the number of those who would take up work in such a disadvantaged school or district is decreasing. In Hungary, for example, as far as the public health care sector is concerned, the dental profession is an aging vocation.^15^ Therefore, dental care, including the range of preventive activities, will become less and less available in these areas, and an increase in health inequality is expected within cumulatively disadvantaged populations.

An American study points to the fact^8^ that the inaccessibility of dental care in disadvantaged groups requires such measures—primarily in the field of prevention—that implementation can only be imagined with the involvement of prevention specialists, increased funding, and greater support from associate professions.^16^

All of this clearly points to the need to take immediate steps to eradicate the deepening social and health inequalities.^11^, ^17^

The aim of our research was to draw attention to the poor oral health indicators of the far-off groups, to find correlations with different health behavior habits, and to offer a solution by exploring the causes of all these.

### Data and methods

Primary school students living in three cumulatively disadvantaged settlements of Borsod-Abaúj Zemplén County and other disadvantaged students living scattered in the county but studying in the same big city high school participated in the data collection of the present research. In the course of the research, we asked them to fill out a dental screening examination, as well as a lifestyle and health behavior questionnaire, and we also used the Rosenberg Self-Assessment Scale^18^ and the Fagerström Nicotine Addiction Test for high school students. With the self-assessment scale, it is possible to learn how young people relate to themselves and their possible health-damaging behavior. In the case of the RSES scale, a higher value indicates greater self-esteem. The Fagerström Nicotine Dependence Test (FTND) can be used to detect nicotine dependence associated with cigarette use. Using this measuring device, one can achieve 0 to 10 points, where a higher score indicates a greater likelihood of physical nicotine dependence. The abbreviated version of the FTND is the Smoking Severity Index, which can be used to more accurately identify strong and weak smokers.^19^ A higher score indicates a greater likelihood of physical nicotine dependence. The evaluation is the following: Up to 2 points: No dependence, 3-4 points: Low dependence, 5-6 points: Moderate dependence, Above 7 points: Strong nicotine dependence.

Among high school students (111 people), smoking was examined with the FTND, which can be used to detect the level of nicotine dependence related to cigarette use. On the scale of the measuring device, 0 to 10 points are available. A value between 0 and 2 points indicates very low dependence, 3 and 4 points indicate low, 5 points indicate medium, 6 and 7 points indicate high, and 8 and 10 points indicate very strong dependence.

The screening test was performed by a dentist using disposable tools. No radiological examination was performed. To assess caries prevalence, we recorded the decayed, missing, and filled teeth (DMFT) values accepted by the WHO (1997), where the DMFT value is the sum of the number of DMFT in relation to the population. The questionnaire was filled out with the help of surveyors after we encountered serious reading and comprehension difficulties among the students.

#### Characterization of the sample

In the study, 429 (318 primary school + 111 high school) young people took part, with an average age of 11.5 (SD: 3.2) years. The youngest was 6 years old, and the oldest was 21 years. Out of those who participated in the data collection, 54.5% were girls and 45.5% were boys.

### Results

#### The socioeconomic background/eating habits

A third of the students live in crowded apartments without comfort, with 33% saying that there is no running water in their apartments (Table 1) These young people have an average of 2.6 siblings, which means that they live in large families (17.5% have two, 68.3% have three or more siblings). A quarter of the students have parents who do not work. Based on the responses to the questionnaire, 3.9% of the older students (who are, on average, 14 years old) did not attend kindergarten.

**Table 1:** The demographic indicators of the sample are as follows

Participant number	N = 429
Average age	11.5 év (SD:3,2)
Gender	Girls 54% Boys 45.5%
Living conditions: Running water at home	Yes, there is: 64.9% No, there isn't: 33% Missing data: 2.1%
Number of siblings	None: 3.2% One: 11.1% Two: 17.5% Three or more: 68.3%
How many people live together in one household?	mean: 6 (SD: 2)
Labor market status	Dad doesn't work: 25.1% Mom doesn't work: 48.7%
Eats breakfast	Yes: 22.3% No: 77.7%
Eats warm meal at home	Yes: 81.6% Only on weekends: 10.9% No: 7.6%
Smoking (Primary school)	Yes, smokes: 10.1%
Smoking (High school)	Yes, smokes: 44.3%
Alcholol consumption (Primary school)	Yes, consumes alcohol: 9.5%
Alcohol consumption (High school)	Yes, consumes alcohol: 20.3%
Dentist availability on the premises	Yes, available: 39.7% No, not available: 60.3%
Frequency of dentist visits	Only for screening: 20.9% Only when it hurts: 73.8% On a regular basis: 5% Missing data: 0.3%

It turned out that 22.3% of students eat breakfast regularly at home, while 77.7% of them reported that having breakfast is not typical for them. Of them, 81.6% eat hot meals every day, 1.9% only on weekends, and 7.6% only rarely. It is more common in the older age group to consume hot meals only on weekends, but out of them, the younger ones are those who consume hot meals infrequently. They mostly eat various soups (33.5%), with no other food being mentioned here. This is followed by the consumption of meat (28.6%) and pasta (13.8%). Fruit consumption is mostly limited to apples, oranges, tangerines, and bananas. Other types of fruit are also mentioned, but significantly less than those listed above. It also came to light that 11% of older, high school–aged students do not consume any fruit at all.

#### Health behavior habits (exercise, smoking, alcohol, and energy drink consumption)

##### Energy drink consumption

The survey showed that 47.8% of students drink energy drinks, with 25.3% consuming them on a daily basis. On a weekly basis, 19.8% of young people consume energy drinks, while 5.1% do so on a monthly basis. They also claimed that energy drinks are usually preferred by girls.

##### Alcohol consumption

Based on the *voluntary* answers, 18.2% of students consume alcohol daily; 6.3% do not know how often, but regularly; 12.6% every week; 29.4% only when relatives get together; and 33.6% rarely, but consume alcoholic beverages. Alcohol consumption is mostly typical of the older age group, over 15 years of age. However, the consumption rate already jumps at the age of 12 to 13, and we have also met a 7-year-old child who said to have consumed alcohol before. Alcohol consumption among high school students is increasing, with 54.6% of students drinking with varying frequency. Of the students who consume alcohol, 27% do so daily, 21.6% weekly, 24.3% rarely, and 25.7% only at family gatherings.

Girls reported a significantly higher proportion of alcohol consumption than boys.

There is a significant correlation between energy drink and alcohol consumption (*P* < .014).

According to the information on physical activity, 24% of students do not do any sports outside of school. They—together with that 11.3% who are also exempt from physical education classes—make up 35.3% of students who do not do any exercise. It also turned out that physical activity decreases with age because elementary school students are much more active than high school students (67.1% of high school students do not exercise). When we asked about the time interval of movement, 16.5% of the students said they exercise for 30 minutes, while 16.6% said they exercise for about 60 minutes or longer. Most people indicated the following forms of exercise: football (4.2%), cycling (2.5%), and dancing (1.7%). It came to light that the parents of these students do not play sports either. According to their own statements, 21.3% of fathers and 14.4% of mothers play sports, which means that the parental model was not given for the students either. There is a significant correlation between the father's education and playing sports (*P* < .004), as skilled workers and fathers with a high school diploma are more likely to do some kind of physical activity. In the case of mothers, there is no correlation to be found between sports and education (*r* = -0.173).

##### Smoking

The percentage of high school students who smoke was 44.3%. The average FTND value of the smokers in the sample is 6.3 (SD: 2.8), which means that the students already showed a high level of addiction (11.1% of smokers had very low, 9.3% had low, 13% had moderate, 27.8% had high, and 38.9% had very strong addiction). There was no correlation between the existence of smoking and DMFT (*r* = 0.07), but we observed a higher DMFT index average in the case of smokers (Table 2).

**Table 2:** DMFT values in relation to smoking among high school students in the studied population (n = 111)

Smoking	DMFT average
No	6.9
Yes	7.8
Total	7.3

##### Oral hygiene habits

Only 24% of young people do not eat or drink anything after brushing their teeth in the evening, 43.2% sometimes, and 32.3% often consume something after brushing their teeth at the end of the day. No correlation was found (*r* = 0.024), but the DMFT index is higher for those young people who still eat after brushing their teeth in the evening (Table 3).

**Table 3:** The relationship between the DMFT value and the consumption of sugary soft drinks and food after brushing the teeth in the evening in the studied population (n = 429)

"Do you usually eat or drink (syrup, tea, soda) after brushing your teeth in the evening?	DMFT average
No	5.9
Yes, sometimes	6.2
Yes, often	6.2

**Fig. 3:**
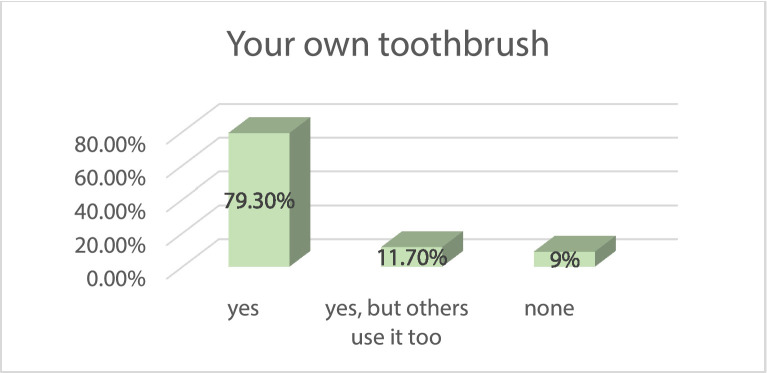
Ownership of own toothbrush among the examined students (n = 429).

According to 8.9% of young people—based on their memories—they did not brush their teeth every day in kindergarten. The current state of oral hygiene is not related to the development of health behavior habits in kindergarten (there is no correlation between DMFT and daily tooth brushing in kindergarten (*r* = 0.025). The percentage of those who have their own toothbrush is 79.3%, while 11.7% have a toothbrush that is also used by someone else, and 9% have no toothbrush at all (Figure 3).

The lack of a toothbrush is more common among younger people under the age of 10 years. The percentage of those who brushed their teeth before school that morning was 69%.

There is a significant, medium-strength correlation between owning a toothbrush and brushing the teeth in the morning (*r* = 0.4). According to their statements, 46.1% of students brush their teeth in the morning and evening, 16.8% every morning, and 10.8% every evening. A total of 13.7% of young people only sometimes brush their teeth, and 7.4% only when they remember. The oral hygiene behavior of the younger age group (10-12 years) is better than the older age group, although there is no significant correlation (*p* > .2); the older age group has a more relaxed attitude towards brushing their teeth frequently. The parental pattern (the health behavior in which the child sees his or her parents brushing their teeth) shows a significant correlation with whether the child brushed his teeth before school in the morning (*p* < .009). We also found that 16.4% of students do not see their parents brushing their teeth.

##### Dental visiting habits

The proportion of those students who said that there is a dentist in their town is 39.7%; however, 60.3% of the children must travel to the dentist because there is no such type of service in their town. A total of 20.9% of young people only go for mandatory screenings, and 73.8% said that they only go when they have a toothache. The number of youngsters who visit the dentist more often and have their teeth filled is minimal. We found that those who brush their teeth regularly in the morning are the ones who visit the dentist more often (*p* < .026).

##### Dental health and gingival state

The percentage of those who had acute gingivitis was 51.6%, while 47.8% had chronic gingivitis (acute, reversible inflammation of the gums, or long-term, irreversible damage) (Table 4). There is no gender difference in gingivitis, but there is a significant correlation with age (*p* < .036). The younger students had acute gingivitis, while the older students had chronic gingivitis. With 13.1% of the young people, we experienced pericoronitis (the gingivitis around the erupting teeth), and in 60.8%, some type of orthodontic or occlusal disorder was observed.

**Table 4:** Gingival state among the examined students (n = 429)

DMFT (mean)	Primary school: 5.08 High school: 7.8
Acute gingivitis	Yes, present: 51.6%
Chronic gingivitis	Yes, present: 47.8%
Tartar	Yes, present: 44%

**Fig. 4:**
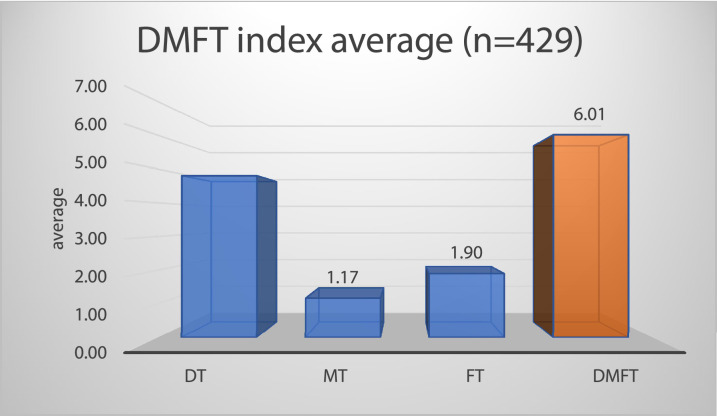
Caries prevalence in the entire sample (sum of decayed “D”, missing “M”, and filled “F” teeth in relation to the studied population) (n = 429).

In the dental screening, 429 students participated. The DMFT mean of permanent teeth is 6 (SD: 3.4) (mean). The minimum index is 0, the maximum is 29. In the case of many students, it can be said that most of their teeth are carious (Figure 4). The students have an average of 4.8 (SD: 3.2) carious teeth (value D). We found a high school student who had 20 carious teeth. On average, 1.1 (SD: 1.02) teeth were missing (M value), and only 1.9 ± 1 teeth were filled (F value).

It is not significant, but boys have worse oral hygiene than girls, with the average DMFT of boys being 6.39, while that of girls is 5.73. There is no correlation between gingivitis and the DMFT index based on the χ^2^ test (*P* < .82). However, a significant correlation exists between the DMFT index and the presence of tartar (*P* < .0001).

**Fig. 5:**
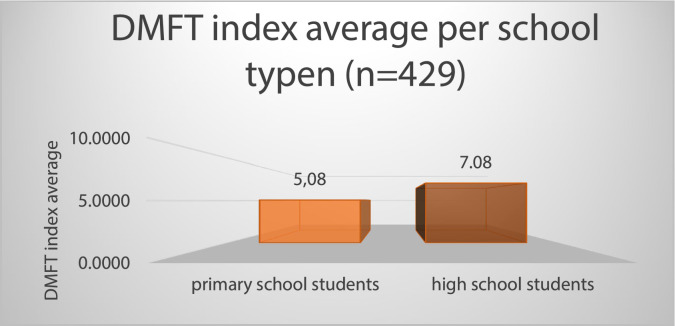
Caries prevalence in the examined group of primary and high school students (n = 429).

There is a weak correlation between alcohol consumption and the DMFT index (*r* = -0.25). Regarding DMFT, there is a significant difference between the school types (*P* < .001) (Figure 5). High school students have significantly worse teeth than elementary school students. So, the dental status of high school students has not improved. The standard deviation of the indices also changes significantly; while that of elementary school students is lower (SD: 3.05), those of secondary school students are significantly higher (SD: 5.6).

Compared to primary school students, the DMFT index is significantly higher in those students who still have a snack after brushing their teeth in the evening (*P* < .005). There is no difference of this kind among high school students (*P* > .5), where the dentition uniformly deteriorates strongly. If we only examine the number of decayed teeth, a significant difference can be found between primary and secondary school students (*P* < .019). While the average D value of elementary school students is 4.7, that of high school students is 6.4.

##### Self-evaluation

A maximum of 40 points can be achieved on the Rosenberg self-assessment scale. The average score of the students participating in the data collection turned out to be 24.9 (SD: 5.9). As far as self-esteem is concerned, it is particularly low (5.6%) in the case of some of the participating students, while 39% have excessive self-esteem. This is also shown by the standard deviation. Lower self-esteem is not related to dental status (*r* = 0.044). Girls’ self-esteem is lower than boys’ (*r* = 0.25, *P* < .05). Morsification was observed in 38.2% of the students (chewing the cheek, substitute act). There is no correlation between self-esteem and the existence of morsification (*r* = -0.14), but it is an interesting fact that this condition occurs to a greater extent in students with higher self-esteem.

## Summary

The studied population consists of young people living in or coming from a sociocultural environment considered to be cumulatively disadvantaged. In the study, we focused on their oral health status, oral hygiene habits, health behavior habits, and in the case of high school students, on self-evaluation and self-esteem that may be associated with these conditions. The analysis of the existing sociocultural background was also an important aspect in terms of their health behavior.

Reducing inequalities and achieving social justice are of prime importance, while attention should also be paid to improving mental and physical health. Identifying and understanding health problems makes it possible to intervene purposefully and bring appropriate development programs to disadvantaged groups because the health awareness of the population living in cumulatively disadvantaged areas is low.^11^

A healthy lifestyle is paramount to promoting health and well-being. This includes proper nutrition, exercise, sufficient rest and sleep, as well as avoiding harmful habits such as smoking or alcohol consumption and also reducing the consumption of one of today's most popular “drugs”, energy drinks.

The individual application of conscious prevention would be extremely important in developing the mental and physical health of young people. It is necessary to raise this to a significantly higher level through regular screenings, inspections, appropriate teaching and motivational activities in disadvantaged populations with the cooperation of suitable professionals for the early recognition of existing health problems and their prevention. In this, the role of education and professional, authentic information is of paramount importance so that specially trained instructors can find the way to the given target community and so that health awareness increases in the community and its members individually recognize the importance of health preservation.

Based on the results, we can conclude that the majority of students studying in the region live in large families (there are 3 or more siblings), with parents of low educational background. A large percentage of them have more than 5 people living in 1 single household. Almost 30% of households with at least three or more children do not have running water, and it is also typical for large families to not have a bathroom. It also came to light that 25% of parents do not work.

Primary and secondary institutions try to organize screenings and programs for children living in disadvantaged regions to preserve and improve the health of young people, but the parental pattern is a determinant in students’ health behavior.^20^ This phenomenon can be clearly observed in alcohol and smoking habits, where 27% of students consume alcohol daily, with 23% doing so at family gatherings. As far as smoking is concerned, the parents of those children who smoke are also heavy smokers.

A large percentage of young people consume energy drinks daily. Regarding smoking, there is a high proportion of smokers and those who would not be able to give up smoking at all in a population with an average age of 16 years. According to HBSC research data, more than 12% of Hungarian high school students smoke, with 57% of smokers doing so daily.^21^ According to another research study conducted in the region, 41.3% of the adult population smokes.^22^ The state of the students’ teeth is also worse than the Hungarian average, but they typically do not feel ashamed because of this (DMFT: 7, in the case of high school students). In the European ranking, Hungary's average DMFT score (2.4) falls within the bottom third of regions (Figure 1). According to the results of this study, the DMFT value for this population in the examined region is significantly worse than the national average**.**^23^

**Figure:**
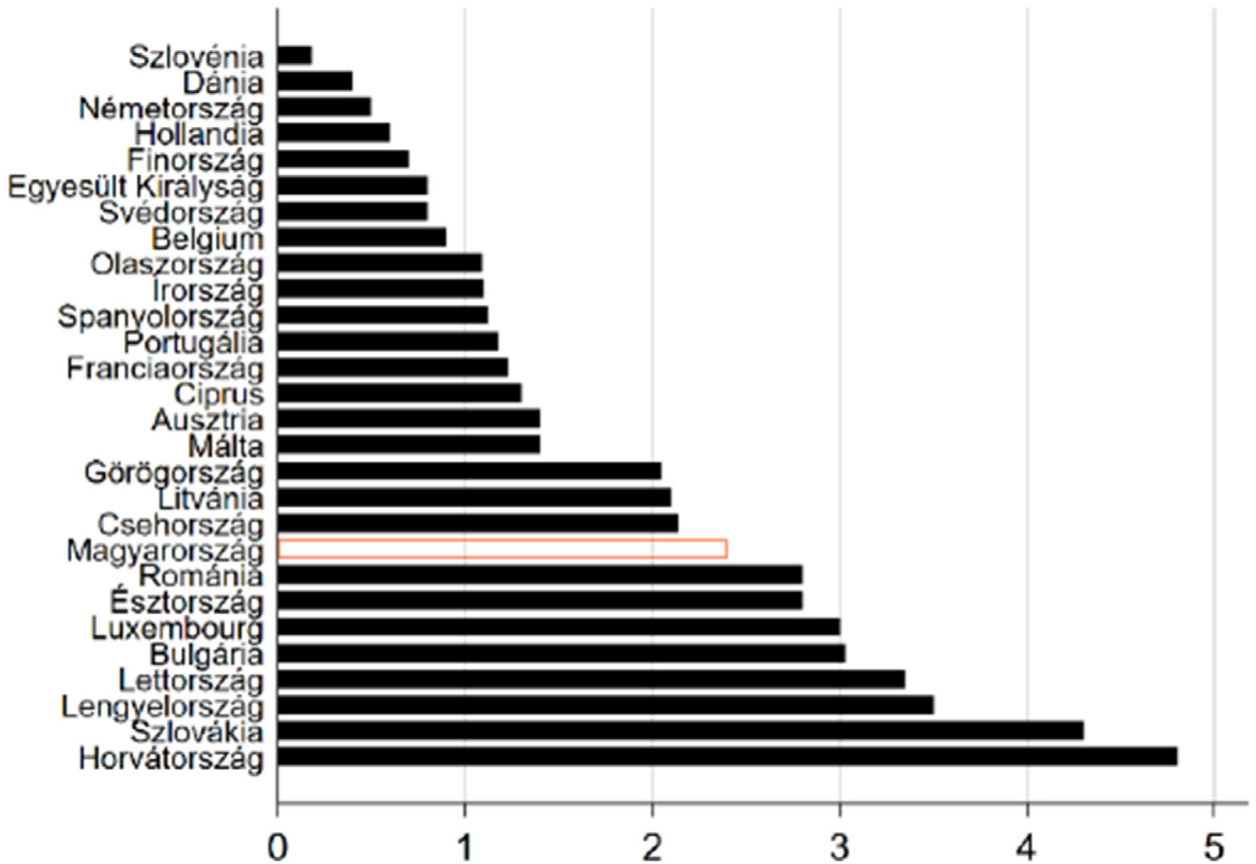
Figure 1. Decayed, missing, and filled teeth value averages in the European Union countries in the 12-year age group.^23^

**Figure:**
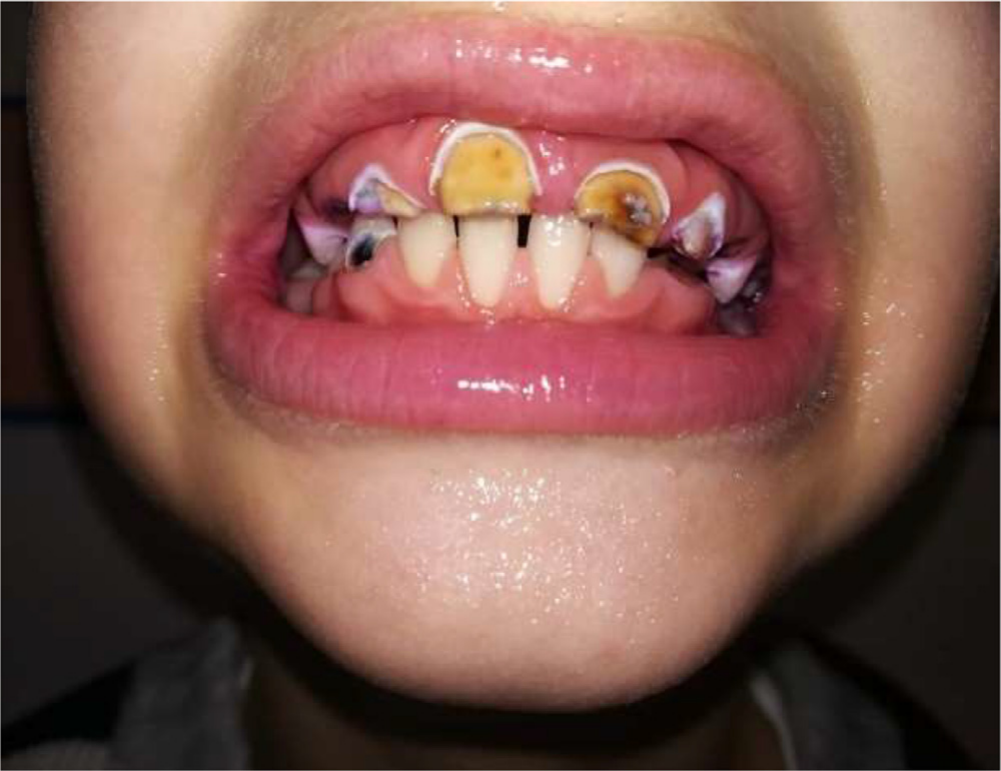
Figure 2. Seven-year-old boy's permanent frontal teeth, from primary school of the studied population.

**Figure:**
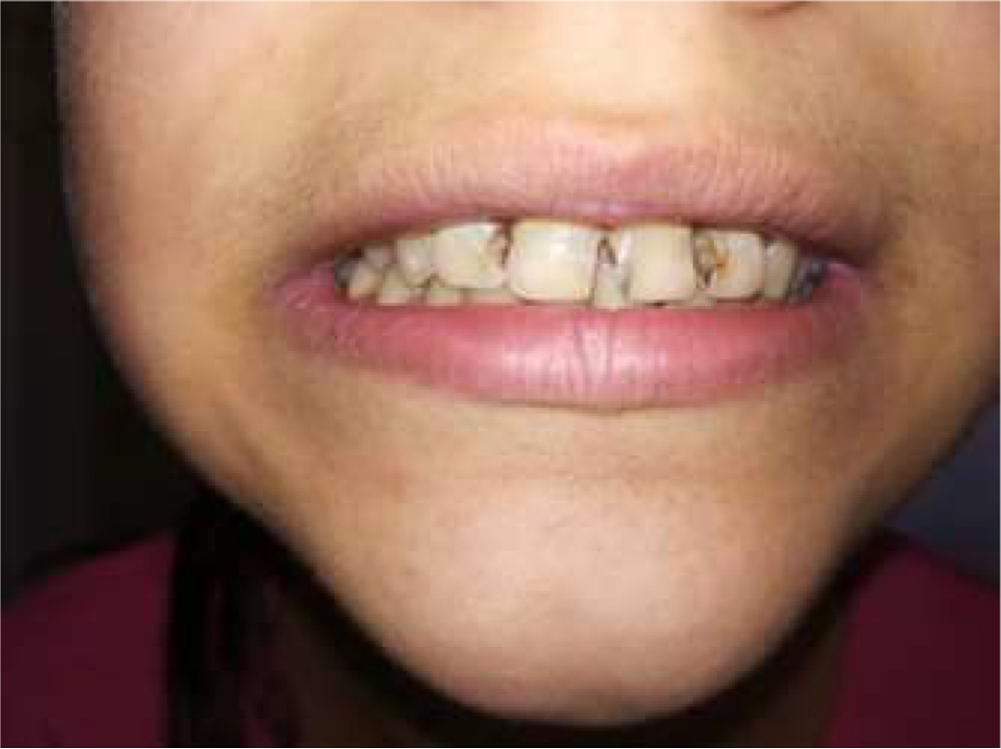
Figure 3. Twelve-year-old boy's decayed frontal teeth, from primary school of the studied population.

The low number of filled teeth indicates the inaccessibility of dental care, as well as the complete lack of preventive activities. The lack of shame associated with bad teeth is a complicating factor as far as prevention in those concerned (Figures 2 and 3).

In contrast to the above, alcohol consumption is related to feelings of shame. Smoking and alcohol are well-known stress relievers, and in a population that does not have adequate coping strategies for the difficulties of everyday life, the high rate of these harmful habits is even more remarkable.

The various harmful health behaviors are even more important in the cumulatively disadvantaged groups, since those diseases that are common in Hungary (such as cardiovascular diseases, pulmonary diseases such as asthma or lung tumors, oral cavity tumors, dental problems) appear more prominently and at a significantly younger age in this social group.

### Conclusions

Since the examined population's visiting habits at the dentist are characterized by the desire to quickly eliminate pain—without participating in any kind of preventive activities—it is easy to see the need to raise the level of health education and health promotion. To improve the oral health and health behavior indicators, which are well below the European average, it is necessary to urgently implement complex public prevention programs with the help of specially trained professionals whose prevention activities include health education, preventive care, and the knowledge and application of psychopedagogical methods. All of this presupposes the launch of new courses, and a significant increase in the financial and moral esteem of the professionals who have graduated there must also be an absolute priority for the sake of effective work.

## Author contributions

Ildikó Faragó: conceptualization and design of the study, writing-original draft. Tímea Egri: acquisition and analysis of data, writing-review, and editing of manuscript. Mihály Kovács: conceptualization and design of the study, writing-review, and editing of manuscript. Andrea Rucska: acquisition and analysis of data, writing-original draft. All authors have approved the final article.

## Conflict of interest

None disclosed.
